# Comparison of Temperature Changes during Implant Osteotomy: Conventional, Single, and Osseodensification Drilling

**DOI:** 10.7150/ijms.105544

**Published:** 2025-02-18

**Authors:** Zeynep Afra Akbiyik Az, Gulsum Ak

**Affiliations:** Istanbul University, Faculty of Dentistry, Department of Oral and Maxillofacial Surgery, Istanbul, Türkiye.

**Keywords:** conventional drilling, implant osteotomy, osseodensification drilling, single drilling, temperature change

## Abstract

**Purpose:** This study compared temperature changes during implant osteotomies using osseodensification drilling (OD), conventional drilling, (CD) and single drilling (SD) protocols on artificial bone blocks of varying densities.

**Methods:** In this *in vitro* study, 240 osteotomies were performed (80 each for CD, SD, and OD protocols) across four bone densities (D1, D2, D3, and D4) and two drilling speeds (800 rpm and 1600 rpm). A drill length of 12 mm and diameter of 3.3 mm were used under irrigated conditions. Temperature changes were measured using an infrared thermal camera, noting the initial temperature (T_0_), the maximum temperature reached (T_1_), and the temperature change (ΔT) before and after each osteotomy, with comparisons drawn among the three protocols.

**Results:** Significant differences in ΔT were observed between the CD and SD protocols across all bone densities and speeds. The CD protocol showed lower ΔT levels at 1600 rpm compared to 800 rpm, whereas the SD protocol showed significantly higher mean ΔT levels at 1600 rpm. For the OD protocol, there was no significant difference in ΔT between the two speeds for D1-D3 densities; however, a significant increase in ΔT was recorded at 1600 rpm for D4. Moreover, the CD protocol consistently yielded the lowest temperature increases in denser bones (D1-D2) at both speeds, while the OD protocol had lower ΔT in less dense bones (D4).

**Conclusions:** The CD protocol consistently achieved lower temperature changes, particularly in denser bones, compared to the SD and OD protocols. The OD protocol, meanwhile, was more effective at reducing temperature increases in less-dense bones.

## Introduction

Atraumatic preparation of implant sites is essential for the success of dental implants 1. Temperature changes during such preparation are critical to osseointegration and implant survival. Excessive temperature increases can cause bone necrosis, potentially delaying osseointegration and increasing the risk of implant failure 2,3.

During surgical preparation of bone tissue, much of the energy used is converted into heat due to friction from drilling. This heat generation poses a risk of bone necrosis, adversely affecting the long-term prognosis of the implant by compromising its primary stability 4,5. Previous studies, both *in vivo* and *in vitro*, have identified several factors contributing to implant failure during osteotomy, such as irrigation method, drill rotation speed, drill diameter and length, drill sharpness, applied force, osteotomy time, drill design and material, and surgical technique 6-9. Furthermore, Soldatos *et al.* investigated temperature dynamics in relation to rotation mode (clockwise, CW, vs. counterclockwise, CCW) in human cadaver tibiae 10.

Augustin *et al.* 9 reported a direct correlation between increased drill rotation speeds and temperature increases in bone, while Gehrke *et al.* 6 found that longer drills tend to produce higher temperatures in bone during osteotomy. Similarly, Soldatos *et al.* reported reduced temperature changes with larger drill diameters in a study using cadaver models 10. Additionally, Möhlhenrich *et al.* 8 noted that higher bone densities are associated with increased temperatures. Despite these insights, few studies have investigated how different implant osteotomy protocols influence temperature changes in peripheral bone during osteotomy 11. Furthermore, no comparative studies have assessed the impact of the osseodensification drilling (OD) protocol across various bone densities.

We studied the impact of the OD protocol on temperature changes during osteotomies in bone blocks of varying densities, comparing it to conventional drilling (CD) and single drilling (SD) protocols. We tested the hypothesis that OD results in lower temperature increases in low-density bones compared to the other protocols.

## Materials and Methods

### Study design

**Figure 1:**
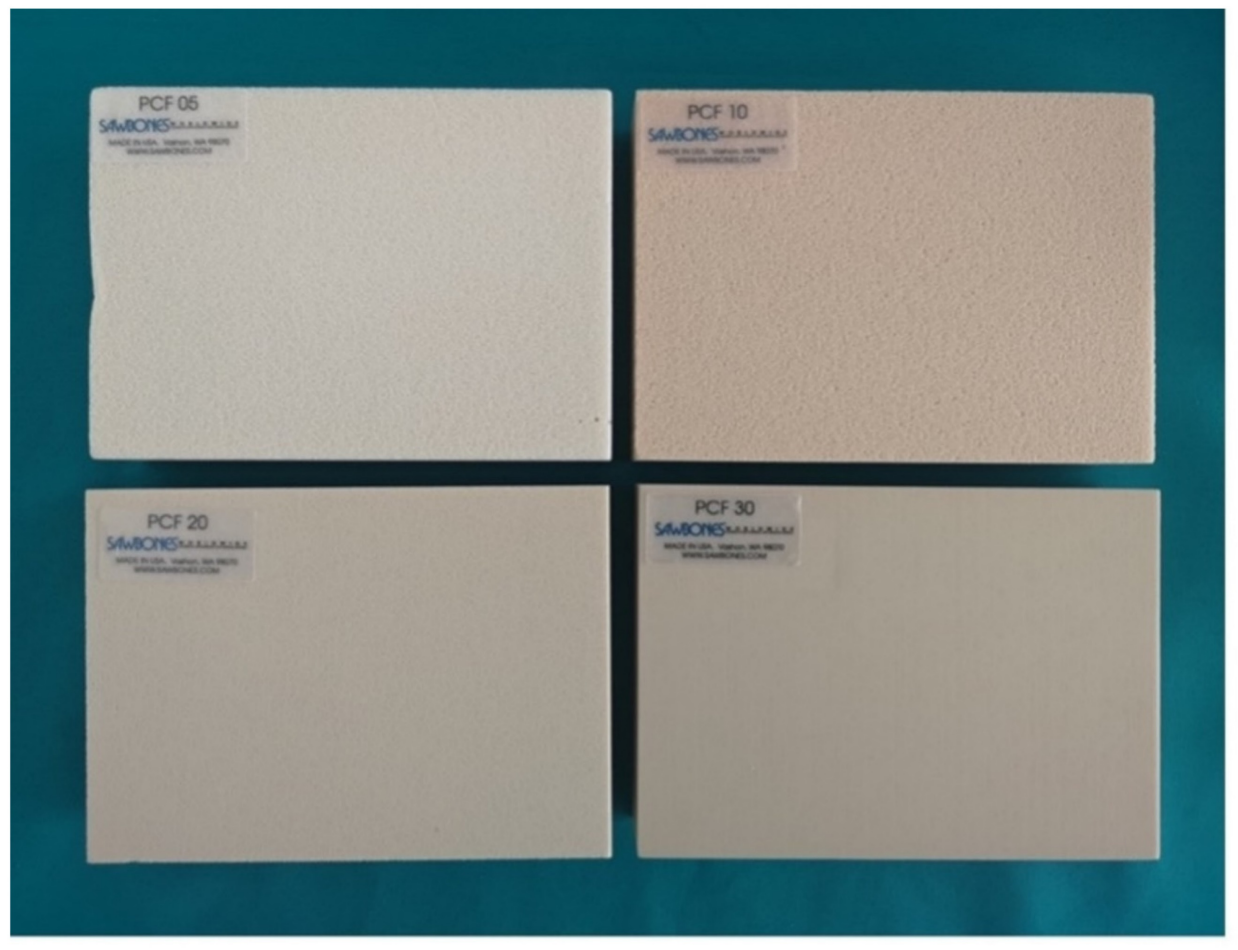
Artificial bone blocks of four different densities (D1-D4).

In this *in vitro* study, implant osteotomies were performed on four different artificial bone blocks: #1522-04 (D1:30 PCF), #1522-03 (D2: 20 PCF), #1522-01 (D3: 10 PCF), and #1522-23 (D4: 5 PCF) (Figure 1), provided by Sawbones (Pacific Research Laboratory Inc., Malmö, Sweden). These blocks are approved by the American Society for Testing and Materials and meet the standards (ASTM F-1839-08) for evaluating orthopedic devices and instruments. We explored temperature variations during bone osteotomy using three different implant osteotomy protocols applied across various bone densities (D1-D4) and two drill speeds (800 rpm and 1600 rpm): CD, SD, and OD. The experiments used a fixed drill length of 12 mm, a drill diameter of 3.3 mm, and irrigated conditions. Temperature changes in the peripheral bone during osteotomy were recorded using an infrared thermal camera.

**Figure 2:**
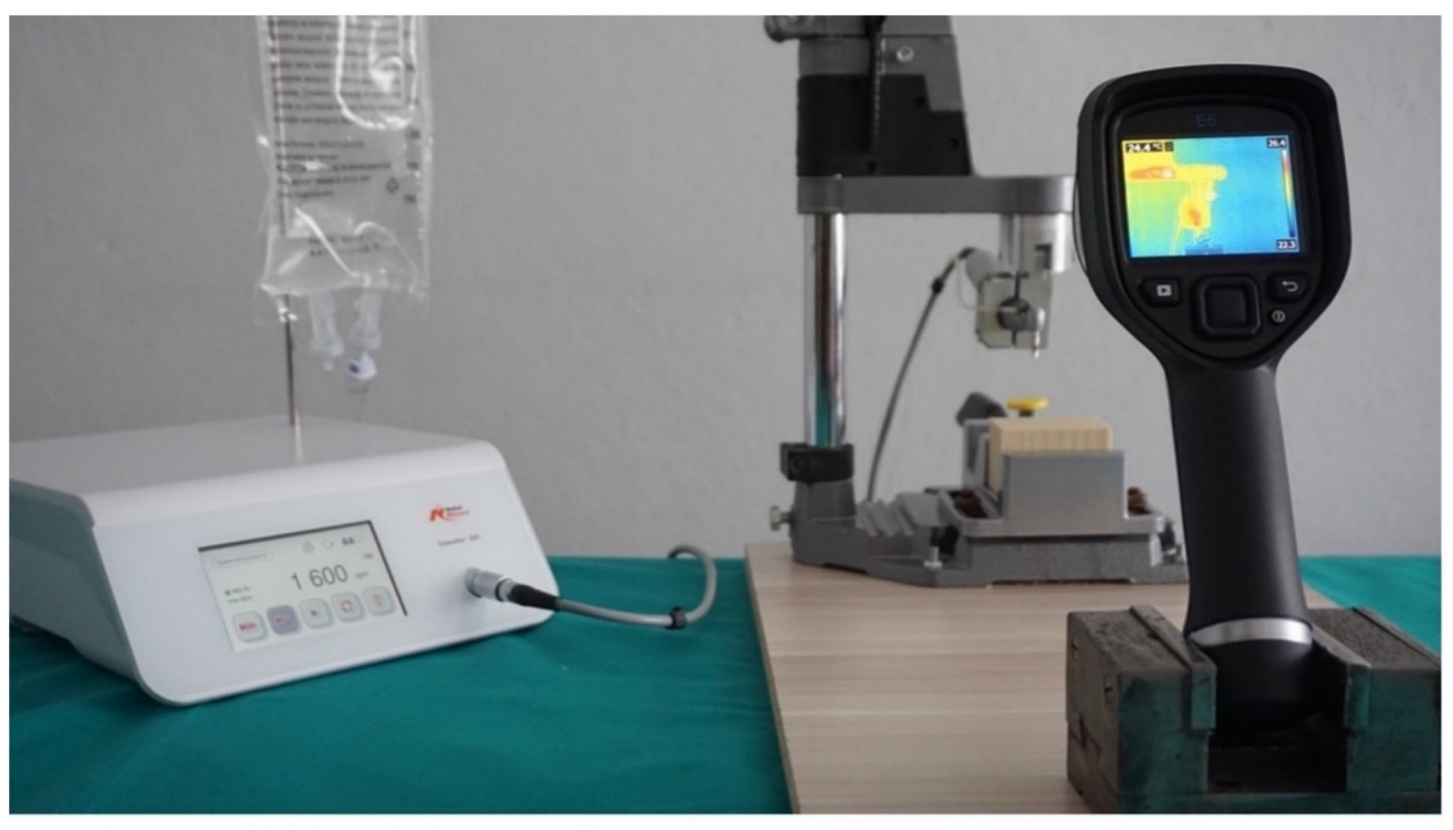
Experimental setup for measuring temperature changes during implant osteotomies.

Experiments were conducted in a controlled room temperature between 20-25°C, under a consistent applied pressure of 2 kg. External irrigation was provided using saline at a steady flow rate of 50 mL/min, also at room temperature. The surgical contra-angle handpiece was secured to the drill stand to maintain the drill at a precise 90° angle to the bone blocks (Figure 2).

The equipment setup included a physiodispenser (Nobel Biocare OsseoSet 300, W&H Dentalwerk, Austria) and a surgical contra-angle handpiece (WS-75L 20:1; W&H, Austria) connected to an implant motor (EM-19LC, W&H, USA). This arrangement was specifically chosen to ensure continuous drilling motion during the osteotomy procedures.

Two examiners participated in this study to ensure consistency and minimize observer bias. Both underwent a calibration process prior to data collection. This process included theoretical training on the study protocols, instruction on the use of equipment, initial practice with standardized bone blocks, repetition trials to minimize temperature changes (ΔT) measurement variability, inter-examiner reliability checks, and a review of evaluation criteria. Examiner calibration was completed at the start of the study using a bone block analog maintained at room temperature (20-25°C). Additionally, practical sessions were conducted, during which the examiners independently assessed pilot osteotomies using the study setup. Calibration was considered successful when all examiners' ΔT readings fell within a 1°C margin of error.

### Osteotomy protocols

The CD osteotomy protocol (Trias Implant System, Servo-Dental GmbH & Co, Hagen, Germany) is a standardized technique for implant site preparation, characterized by the progressive enlargement of the osteotomy site. The protocol begins with a 1.6 mm drill, followed by intermediate drills of ⌀2.0 mm and ⌀3.0 mm, and concludes with final drills of various diameters, including ⌀3.3 mm, ⌀3.8 mm, ⌀4.3 mm, and ⌀5.0 mm.

The SD osteotomy protocol (IBS Implant Magic Core System, InnoBioSurg Co., Ltd, South Korea) uses a one-stage osteotomy protocol, which differs from traditional implant systems. This protocol uses specially grooved drills of specific diameters to prepare the implant site in a single step. The SD protocol includes drills with diameters of ⌀1.6 mm, ⌀2.8 mm, ⌀3.3 mm, ⌀3.8 mm, ⌀4.3 mm, and ⌀4.8 mm.

The OD osteotomy protocol (Versah, Jackson, MI, United States) involves Densah drills that, unlike traditional systems, compress rather than cut bone 12. This is achieved through CCW rotation, pressing the bone against the peripheral tissue and enhancing bone density through autografting during osteotomy. The Densah drills in the Versah OD system come in various diameters, including ⌀1.6 mm, ⌀2.0 mm, ⌀2.3 mm, ⌀2.5 mm, ⌀3.0 mm, ⌀3.3 mm, ⌀3.5 mm, ⌀4.0 mm, ⌀4.3 mm, ⌀4.5 mm, ⌀5.0 mm, ⌀5.3 mm, and ⌀5.5 mm.

The OD protocol was implemented in its CCW rotation mode across all bone densities (D1-D4) to ensure methodological consistency and facilitate direct comparison with the CD and SD protocols. This approach allowed for a standardized evaluation of the thermal dynamics and drilling efficiency of all three systems across varying bone densities.

In this study, we used the ⌀3.3 mm diameter as the final drill and 12 mm length implants for all protocols to ensure consistency and comparability across the systems. This size is commonly used in narrow alveolar ridges and is relevant to clinical scenarios.

### Temperature measurements

**Figure 3:**
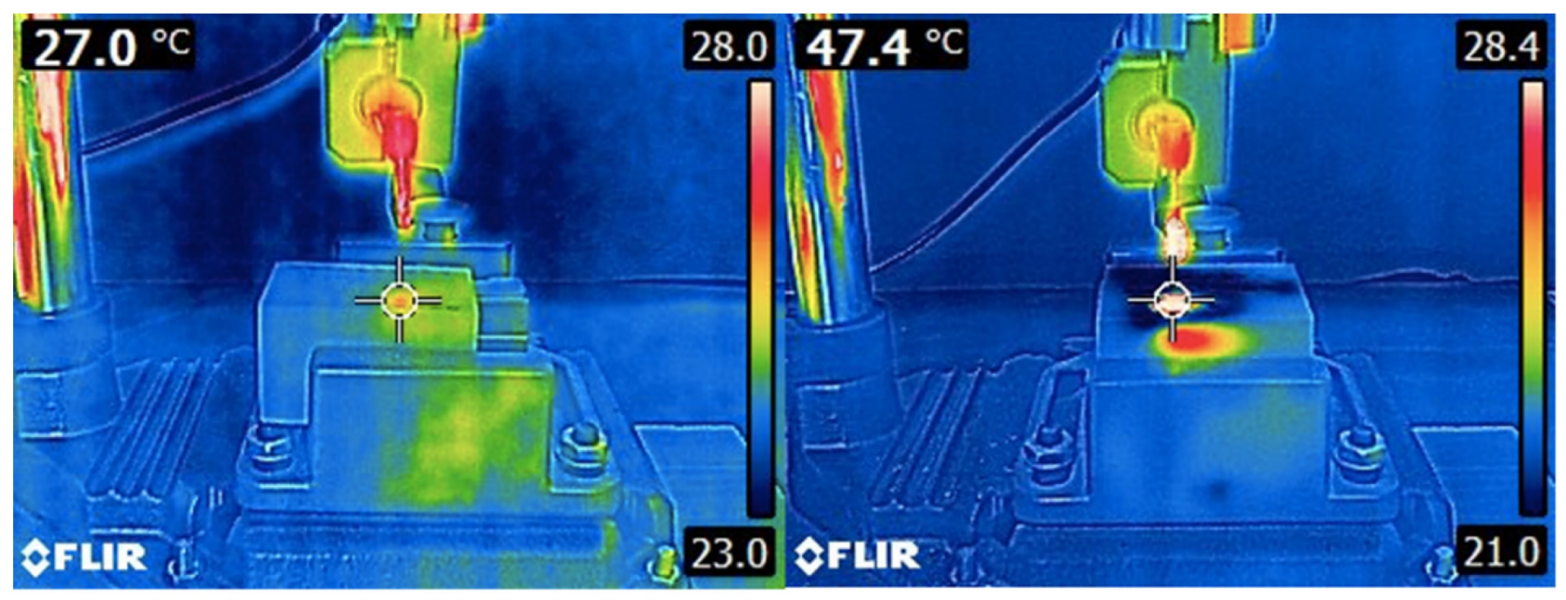
Infrared thermal image illustrating the thermal emission area of a polyurethane block before and after osteotomy, captured with FLIR E6-XT camera.

Thermal image series were captured during implant osteotomy using a 14-bit digital infrared thermal camera (FLIR E6-XT, FLIR Systems OÜ, Estonia). The acquisition parameters for the thermal images were set as follows: a 240 × 180 focal plane array (43,200 pixels), a spectral range of 7.5-13 μm, thermal sensitivity (NETD) < 0.06 °C (0.11°F) / < 60 mK, an image frequency of 9 Hz, and a field of view of 45° × 34°. The infrared thermal camera was positioned 30 cm away from the sample, as recommended by the manufacturer, and mounted on a height-adjustable holder to ensure precise alignment with the sample. This configuration maximized spatial resolution and used FLIR MSX imaging (Multi-Spectral Dynamic Imaging) to optimize recording sensitivity and accuracy (Figure 3). Temperature variation in the artificial bone blocks during implant osteotomy were assessed with these thermal images. For each osteotomy, we recorded the initial temperature (T_0_), the maximum temperature reached in the bone (T_1_), and the ΔT before and after the osteotomy. To prevent heat accumulation during successive procedures, the bone was allowed to return to its initial temperature after each osteotomy.

### Statistical analyses

Data were analyzed using SPSS software (version 27.0 for Windows; IBM Corp., Armonk, NY, USA). Numerical data (T_0_, T_1_, ΔT) are presented as mean ± standard deviation (SD), with minimum and maximum values. The distribution of variables was assessed using the Shapiro-Wilk test. One-way analysis of variance test was used to assess temperature changes among implant protocols (CD, SD, or OD). For normally distributed data, we used ANOVA, followed by Tukey's multiple comparison test for subgroup analyses (CD vs. SD, CD vs. OD, and SD vs. OD). A *p*-value < 0.05 was considered statistically significant.

## Results

Table 1 presents the temperature measurements (T_0_, T_1_, ΔT) across four different artificial bone blocks during drilling, using three osteotomy protocols: CD, SD, and OD. In total, 240 implant osteotomies were conducted, with 60 for each bone density category (D1-D4). The highest mean T_1_ level (41.58°C) was observed in D1 density at a drilling speed of 1600 rpm using the SD protocol, while the lowest mean T_1_ level (21.66°C) was recorded in D3 density at a drilling speed of 800 rpm using the SD protocol. The highest mean ΔT level (18.64°C) was also observed in D1 density at a drilling speed of 1600 rpm using the SD protocol. In contrast, the lowest mean ΔT level (1.06°C) was observed in D4 density at a drilling speed of 800 rpm using the OD protocol.

Table 2 compares the mean ΔT levels during different osteotomy protocols in artificial bone blocks across various bone densities and drilling speeds. Statistically significant differences (*p* < 0.05 unless noted otherwise) were noted between CD and SD protocols for all bone densities (D1-D4) and both drilling speeds (800 rpm and 1600 rpm) in terms of ΔT levels. Additionally, the CD protocol consistently exhibited significantly lower mean ΔT levels at 1600 rpm compared to 800 rpm across all bone densities. Conversely, the mean ΔT levels for the SD protocol were significantly higher at 1600 rpm compared to 800 rpm for all bone densities. For the OD protocol, no significant difference in mean ΔT levels was found between 800 rpm and 1600 rpm for bone densities D1-D3. However, for D4 bone density, a significantly higher mean ΔT level was observed at 1600 rpm compared to 800 rpm.

Table 3 presents a subgroup analysis comparing drilling protocols across various bone densities (D1-D4) and drilling speeds (800 and 1600 rpm). For D1 bone density, the CD protocol demonstrated statistically significant lower temperature increases compared to both SD and OD protocols at both 800 rpm and 1600 rpm (*p* = 0.0001 for all comparisons). Similarly, in D2 bone density, the CD protocol showed statistically significant lower temperature increases than the SD and OD protocols at both speeds (*p* = 0.0001 for all), with the OD protocol showing a lower temperature increase than the SD protocol at 1600 rpm (*p* = 0.013). In D3 bone density, the SD protocol displayed a significantly lower temperature increase compared to the CD and OD protocols at 800 rpm (*p* = 0.0001 for both), while the CD protocol showed a lower temperature increase than the OD (*p* = 0.0020). Furthermore, at 1600 rpm, the OD protocol demonstrated significantly higher temperature increases than both the CD and SD protocols (*p* = 0.0001 and *p* = 0.0010, respectively). In D4 bone density, the OD protocol showed significantly lower temperature increases compared to the CD and SD protocols at both 800 rpm and 1600 rpm.

## Discussion

Previous research has identified several factors affecting bone preparation, including irrigation technique, drill shape, drilling depth, drill diameter, and drill sharpness. However, no prior studies have compared the temperature changes during implant osteotomy among the OD protocol and the SD and CD protocols. This study compared temperature changes among three implant osteotomy protocols and assessed the effects of drill rotation speed during implant osteotomy. We noted lower mean ΔT values in the CD protocol at higher drill speeds across all bone densities, no significant differences in mean ΔT values between drill speeds for bone densities D1-D3 in the OD protocol, significantly lower temperature changes in high-density bones using the CD protocol compared to SD and OD at both speeds, and superior performance of the OD protocol in maintaining lower ΔT increases in D4-density bones compared to the other protocols.

*Ex vivo* studies often use bovine and porcine bone models, but the internal structure of these bones can vary widely among specimens. Porcine and bovine bones exhibit densities similar to those classified as D3 or D4 13,14. Similarly, polyurethane bone blocks with a density of 20 PCF and a cortical layer of 3 mm at 50 PCF closely mimic the osteotomy temperatures and times of human rib bones 15. While cadaveric bones more accurately represent human mandibular bone in terms of structural and thermal properties, their heterogeneity introduces challenges for controlled experiments. To reduce variability and standardize results, we used polyurethane-based bone blocks. The choice of artificial bone blocks allowed for greater reproducibility and standardization by eliminating the variability inherent in human or animal cadaver bones, such as differences in density, cortical thickness, and trabecular structure.

Research on real-time temperature increases during osteotomy has used indirect methods such as infrared thermography and direct methods such as thermocouples. While thermocouples measure temperature directly through heat-sensitive probes, they are influenced by factors such as probe isolation, recording depth, sensor materials, and measurement errors 16. In contrast, infrared thermography provides an indirect measurement of surface temperatures by creating a thermal profile of the drill and surrounding tissue. Infrared thermography provides a more sensitive and accurate assessment of intraosseous temperature changes during osteotomy than thermocouples 17. Moreover, although infrared thermography is susceptible to the effects of liquids such as saline, the controlled flow rate and room temperature of the saline utilized in this study minimized any potential interference, thereby ensuring the accuracy of the temperature measurements. Previous studies have also successfully employed infrared thermal cameras for evaluating temperature changes during osteotomy, emphasizing their reliability in similar experimental setups 8, 16. Based on these considerations, we used infrared thermography in this study to obtain the most reliable temperature data.

The selection of 800 and 1600 RPM as the drilling speeds in this study was based on their representation of clinically relevant speed ranges and alignment with recommendations from implant manufacturers. Eriksson *et al.* recommended speeds of 1500-2000 RPM under irrigation to reduce thermal injury 18, while Sharawy *et al.* demonstrated that higher speeds could mitigate heat generation by minimizing drilling time 19. The selection of 800 and 1600 RPM as the drilling speeds in this study was based on the rationale that these represent clinically relevant speed ranges and align with recommendations from implant manufacturers. However, it should be acknowledged that the exclusion of intermediate speeds, such as 1000 and 1100 RPM, introduces a limitation to the study design. Consequently, it is recommended that future studies include these speeds to provide a more comprehensive evaluation of their effects on thermal changes during osteotomy.

Higher drill rotation speeds during implant osteotomy typically result in increased temperatures due to elevated friction. Augustin *et al.* 9 reported a positive correlation between drill speed and increases in bone temperature in an *in vitro* study, while Delgado-Ruiz *et al.* 20 observed significantly lower temperatures at slower drill speeds while investigating the SD protocol. Similarly, we observed greater changes in temperature at higher speeds using the SD protocol. In contrast, the CD protocol consistently showed lower temperature changes at higher speeds across all bone densities. Sharawy *et al.* noted that higher speeds in the CD protocol reduced both osteotomy time and heat generation, especially in dense bones, suggesting that increased speeds minimize thermal exposure by shortening drilling time 19. Two other studies also reported that higher drill rotation speeds can reduce temperature changes during osteotomy 21,22. However, our findings showed no significant temperature differences within the OD protocol across various drill speeds in bone densities D1-D3. The OD protocol exhibited a more stable temperature response to increasing speeds compared to the SD protocol. These results underscore the complex relationship between drill rotation speeds and temperature changes during implant osteotomy, highlighting protocol-specific variations and providing valuable insights into the temperature dynamics of the OD protocol, which had not been previously evaluated in a similar context.

We also found that the CD protocol consistently yielded lower temperature changes in dense bone compared to the SD and OD protocols, corroborating previous findings 23-25. The gradual, stepwise progression of the CD protocol allows the initial perforation to establish a pathway, facilitating subsequent drill advancements that minimally elevate the temperature with each step 26. In contrast, the SD protocol involves removing the entire volume of bone in a single step, which increases friction and heat generation during osteotomies at the final diameters. This increased heat generation is particularly pronounced in dense bone. Conversely, in low-density bone with a trabecular structure, reduced friction and heat generation lead to a lower thermal impact. Although significant differences were identified among the osteotomy protocols, the mean ΔT values among the three systems were closely aligned in D3-density bone, indicating comparable thermal responses under these specific conditions.

No previous studies have evaluated the temperature changes in bone during implant osteotomy using the OD protocol compared to other osteotomy protocols. Our findings indicate that although the OD protocol results in higher temperature increases than both the CD and SD protocols in dense bones, it produces the lowest temperature increases in D4 bone density. The OD protocol, distinct from the CD and SD protocols, compresses and condenses the bone during osteotomy, converting it into an autogenous graft that enhances the existing bone's density 27,28. Consequently, higher temperatures are inevitable, particularly in dense bones. However, the densification process of the OD protocol significantly enhances the quality and quantity of autologous bone, contributing to the primary stability of the implant 29,30, especially in low-density bones like D4, without causing an additional rise in temperature. Similarly, Soldatos *et al.* noted that the CCW mode, which corresponds to the OD protocol in our study, can enhance bone density in low-density bones while generating lower temperature increases, thereby creating an autograft effect that supports implant primary stability 10. However, Soldatos *et al.* found that repeated use of drills more than 23 times in CCW mode could lead to excessive temperature increases, potentially causing bone necrosis 10. In contrast, our findings indicate that temperature changes in the OD protocol were primarily influenced by bone density rather than drill reuse frequency, as higher ΔT values were observed in denser bone types (D1-D3), even without exceeding a specific drill usage threshold. However, our study demonstrated that the CCW mode provided the lowest temperature increases in low-density bones (D4). Nevertheless, our study demonstrated that the CCW mode resulted in the lowest temperature increases in low-density bones (D4), reinforcing the advantage of the OD protocol in maintaining thermal stability while simultaneously improving bone properties in less-dense regions.

Our study had several limitations. The primary limitation was its *in vitro* design. The synthetic bone blocks used exhibit uniform physical properties to standardize procedures and minimize measurement deviations. However, due to the inherent variability in human jaw bones, there may be discrepancies between this model and actual *in vivo* conditions. Moreover, *in vitro* bone simulations lack blood circulation and physiological body temperature, factors critical for heat dissipation. Additionally, the cooling process via irrigation may present a challenge for infrared thermography, potentially obscuring temperature measurements in the deeper layers of the osteotomy site. However, this potential issue was addressed by meticulously regulating the flow rate and temperature of the saline solution during the experimental procedures. This approach ensured a consistent irrigation environment and minimized the impact of surface temperature readings.

## Conclusions

The CD protocol consistently resulted in lower temperature changes, particularly in dense bones, compared to the SD and OD protocols. Additionally, while the OD protocol showed the highest temperature increases in dense bones, it was most effective in low-density bones, maintaining the lowest temperature increase. These findings highlight the importance of developing drilling protocols that are tailored to specific bone densities and clinical environments. Despite the higher temperatures observed with the OD protocol in high-density bones, its ability to improve bone quality and stability makes it beneficial for use in low-density bones. Further *in vivo* research is needed to determine if these findings are applicable to human subjects, which would help in establishing the most effective drilling protocols for different bone types.

## Figures and Tables

**Table 1:** Temperature measurements (T_0_, T_1_, ΔT) in four different artificial bone blocks during drilling with three osteotomy protocols

			Conventional drilling			Single drilling		Osseodensification drilling	
	Drilling speed	Temp.	mean ± SD	(min - max)	mean ± SD		(min - max)	mean ± SD	(min - max)
**D1**	800 rpm	T_0_	21.40 ± 0.45	(20.70 - 21.90)	24.16 ± 0.62		(23.20 - 24.20)	23.81 ± 0.58	(22.90 - 24.90)
		T_1_	24.36 ± 0.49	(23.40 - 24.80)	38.52 ± 3.79		(34.00 - 38.00)	41.16 ± 3.88	(34.00 - 47.50)
		ΔT	2.96 ± 0.64	(2.00 - 4.10)	14.36 ± 3.52		(9.10 - 14.10)	17.35 ± 4.05	(10.00 - 23.90)
	1600 rpm	T_0_	21.37 ± 0.59	(20.50 - 22.30)	22.94 ± 0.93		(21.00 - 22.00)	24.34 ± 0.65	(23.30 - 25.50)
		T_1_	23.44 ± 1.21	(21.50 - 24.80)	41.58 ± 2.88		(37.50 - 41.50)	40.91 ± 3.23	(34.00 - 45.50)
		ΔT	2.07 ± 0.79	(0.60 - 2.90)	18.64 ± 3.30		(13.20 - 18.20)	16.57 ± 3.29	(9.70 - 22.10)
**D2**	800 rpm	T_0_	20.59 ± 0.44	(19.90 - 21.40)	21.94 ± 0.38		(21.40 - 22.50)	23.57 ± 1.33	(21.40 - 25.80)
		T_1_	23.36 ± 0.45	(22.50 - 24.10)	30.38 ± 3.10		(26.40 - 34.80)	33.07 ± 3.17	(28.30 - 39.80)
		ΔT	2.77 ± 0.47	(2.10 - 3.50)	8.44 ± 3.05		(4.90 - 12.50)	9.50 ± 2.82	(6.90 - 16.60)
	1600 rpm	T_0_	21.79 ± 0.30	(21.40 - 22.40)	22.57 ± 0.40		(21.80 - 23.20)	24.35 ± 1.11	(22.80 - 26.40)
		T_1_	24.09 ± 0.20	(23.60 - 24.30)	37.99 ± 2.86		(34.20 - 42.50)	36.05 ± 4.17	(31.90 - 44.10)
		ΔT	2.30 ± 0.29	(1.90 - 2.80)	15.42 ± 2.73		(11.60 - 19.90)	11.70 ± 3.83	(7.00 - 19.20)
**D3**	800 rpm	T_0_	21.74 ± 0.24	(21.40 - 22.20)	19.98 ± 0.43		(19.40 - 20.70)	22.02 ± 0.27	(21.60 - 22.50)
		T_1_	23.71 ± 0.22	(23.30 - 24.10)	21.66 ± 0.23		(21.30 - 22.00)	25.81 ± 0.40	(25.20 - 26.50)
		ΔT	1.97 ± 0.36	(1.30 - 2.70)	1.68 ± 0.39		(0.90 - 2.30)	3.79 ± 0.41	(3.30 - 4.50)
	1600 rpm	T_0_	21.33 ± 0.48	(20.40 - 21.90)	20.64 ± 0.54		(19.50 - 21.40)	22.18 ± 0.20	(21.90 - 22.50)
		T_1_	23.64 ± 0.32	(23.10 - 24.00)	23.28 ± 0.42		(22.50 - 23.90)	25.95 ± 0.36	(25.60 - 26.60)
		ΔT	2.31 ± 0.31	(1.80 - 2.70)	2.64 ± 0.52		(2.00 - 3.50)	3.77 ± 0.38	(3.40 - 4.60)
**D4**	800 rpm	T_0_	22.38 ± 0.28	(21.90 - 22.90)	21.77 ± 0.57		(21.00 - 23.10)	24.33 ± 0.36	(24.50 - 24.90)
		T_1_	24.58 ± 0.13	(24.30 - 24.80)	23.78 ± 0.11		(23.60 - 24.00)	25.39 ± 0.33	(25.80 - 25.80)
		ΔT	2.20 ± 0.25	(1.70 - 2.60)	2.01 ± 0.60		(0.70 - 3.00)	1.06 ± 0.27	(1.60 - 1.40)
	1600 rpm	T_0_	23.06 ± 0.38	(22.50 - 23.60)	21.44 ± 0.28		(21.00 - 21.70)	22.94 ± 0.70	(22.00 - 24.20)
		T_1_	24.78 ± 0.21	(24.50 - 25.10)	24.13 ± 0.24		(24.00 - 24.80)	24.46 ± 0.25	(24.20 - 25.00)
		ΔT	1.72 ± 0.36	(1.00 - 2.30)	2.69 ± 0.43		(2.40 - 3.80)	1.52 ± 0.61	(0.80 - 2.70)
									

**Note:** Data are expressed as numbers (n), mean, standard deviation (SD), minimum, and maximum values. **Abbreviations:** T_0_, temperature level on admission; T_1_, temperature level at final; ΔT, temperature change

**Table 2:** Comparative analysis of the temperature difference values (ΔT) observed during different drilling protocols in artificial bone blocks with varying bone densities and drilling speeds

		Conventional Drilling	Single Drilling	Osseodensification Drilling	p-value
Bone density	Drilling speed	mean ± SD	mean ± SD	mean ± SD	
**D1**	800 rpm	2.96 ± 0.64	14.36 ± 3.52	17.35 ± 4.05	0.0001
	1600 rpm	2.07 ± 0.79	18.64 ± 3.30	16.57 ± 3.29	0.0001
	**p-value**	0.0070	0.0120	0.6420	
**D2**	800 rpm	2.77 ± 0.47	8.44 ± 3.05	9.50 ± 2.82	0.0001
	1600 rpm	2.30 ± 0.29	15.42 ± 2.73	11.70 ± 3.83	0.0001
	**p-value**	0.0030	0.0001	0.1600	
**D3**	800 rpm	2.31 ± 0.31	1.68 ± 0.39	3.79 ± 0.41	0.0001
	1600 rpm	1.97 ± 0.36	2.64 ± 0.52	3.77 ± 0.38	0.0001
	**p-value**	0.0130	0.0001	0.9110	
**D4**	800 rpm	2.20 ± 0.25	2.01 ± 0.60	1.06 ± 0.27	0.0010
	1600 rpm	1.72 ± 0.36	2.69 ± 0.43	1.52 ± 0.61	0.0010
	**p-value**	0.0150	0.0010	0.0350	

**Note:** Data are expressed as numbers (n), mean, and standard deviation (SD). One-way analysis of variance test was used to assess temperature changes among implant protocols (CD, SD, or OD).

**Table 3:** Subgroup analysis of drilling protocol comparisons across bone densities and drilling speeds

		Bone Density			
	Drilling speed	D1	D2	D3	D4
**CD vs. SD**	800 rpm	0.0001	0.0001	0.0001	0.0040
	1600 rpm	0.0001	0.0001	0.0950	0.0020
**CD vs. OD**	800 rpm	0.0001	0.0001	0.0020	0.0030
	1600 rpm	0.0001	0.0001	0.0001	0.0098
**SD vs. OD**	800 rpm	0.1000	0.5910	0.0001	0.0020
	1600 rpm	0.2180	0.0130	0.0010	0.0020

**Note:** Subgroup analyses (CD vs. SD, CD vs. OD, and SD vs. OD) were conducted using Tukey multiple comparison test. **Abbreviations:** CD, conventional drilling; SD, single drilling; OD, osseodensification drilling
